# Differential Effects of Senescence on the Phloem Exports of Cadmium and Zinc from Leaves to Grains in Rice during Grain Filling

**DOI:** 10.3390/plants12091902

**Published:** 2023-05-06

**Authors:** Chengfeng Hu, Bofang Yan, Yating Liu, Chen Gong, Man Zhao, Rongliang Qiu, Yetao Tang

**Affiliations:** 1 Guangdong Provincial Key Laboratory of Environmental Pollution Control and Remediation Technology, School of Environmental Science and Engineering, Sun Yat-Sen University, Guangzhou 510275, Chinaeesqrl@mail.sysu.edu.cn (R.Q.); 2 Guangdong Laboratory for Lingnan Modern Agriculture, Guangzhou 510642, China; 3 Guangdong Provincial Key Laboratory of Agricultural & Rural Pollution Abatement and Environmental Safety, College of Natural Resources and Environment, South China Agricultural University, Guangzhou 510642, China

**Keywords:** food safety, biofortification, source tracing, subcellular distribution, transporter, isotope labeling

## Abstract

In rice, non-essential toxic cadmium (Cd) and the essential nutrient zinc (Zn) share similar transport pathways, which makes it challenging to differentially regulate the allocation of these elements to the grain. The phloem is the main pathway for the loading of these elements into rice grains. It has long been accepted that tissue senescence makes the nutrients (e.g., Zn) stored in leaves available for further phloem export toward the grain. Whether senescence could drive the phloem export of Cd remains unclear. To this end, the stable isotopes ^111^Cd and ^67^Zn were used to trace the phloem export and the subsequent allocation of Cd and Zn from the flag leaves, where senescence was accelerated by spraying abscisic acid. Furthermore, changes upon senescence in the distribution of these elements among the leaf subcellular fractions and in the expression of key transporter genes were investigated. Abscisic acid-induced senescence enhanced the phloem export of Zn but had no impact on that of Cd, which was explained by the significant release of Zn from the chloroplast and cytosol fractions (concentrations decreased by ~50%) but a strong allocation of Cd to the cell wall fraction (concentration increased by ~90%) during senescence. Nevertheless, neither Zn nor Cd concentrations in the grain were affected, since senescence strengthened the sequestration of phloem-exported Zn in the uppermost node, but did not impact that of phloem-exported Cd. This study suggests that the agronomic strategies affecting tissue senescence could be utilized to differentially regulate Cd and Zn allocation in rice during grain filling.

## 1. Introduction

Mineral levels in cereals are critical for food safety and nutrition, among which the levels of cadmium (Cd) and zinc (Zn) are of particular concern [1,2]. For both higher plants and humans, Cd is a non-essential and toxic metal that could cause metabolic disorders, whereas Zn is an essential nutrient that is fundamental to enzyme activities [3,4]. Among the world’s population, especially the Asian population, rice accounts for a major portion of the caloric intake [5]. However, rice is characterized as a major source of human exposure to Cd but a poor source of dietary Zn [2,6]. Hence, Cd accumulation must be diminished and Zn accumulation be fortified in rice grains, in order to prevent the population from experiencing Cd toxicities and Zn deficiencies. This requires a further understanding of the allocation processes of these elements in rice plants.

Owing to their similar chemical properties, Cd and Zn transport processes from soil to grains are generally similar, resulting in a simultaneous reduction of Zn in grains when agronomic measures are taken to reduce Cd in grains [7,8,9]. Thus far, it remains challenging to differentially regulate Cd and Zn accumulations in rice grains [10]. In acidic soils, both the solubility of Cd and Zn and the subsequent uptake decrease with an increase in soil pH, since the higher pH causes weaker ion–proton exchange and thereby stronger and nonspecific adsorption of Cd and Zn onto the negatively charged solid phase [7,11]. In plants, it is well known that Cd shares transporters with Zn, iron (Fe) and manganese [6]. For example, it was found that both Zn and Cd can be taken up by OsIRT1 in rice roots [12], where OsHMA3 mediates vacuole compartmentalization while OsHMA2 is involved in the xylem loading of both elements for root-to-shoot translocation [1,13]. Both Cd and Zn are translocated in the xylem mostly as free ions [14,15] and highly allocated toward the photosynthetic organs (e.g., leaves) with the transpiration flow during the vegetative growth period [16,17]. During grain filling, OsHMA2 in the node facilitates the allocation of both Cd and Zn to the grain by mediating the intervascular transport of these elements [18]. Together, these similarities make the Cd and Zn content in grains always positively correlated. Indeed, some studies found that applying lime reduces the soil-available Cd and grain Cd concentrations, but also reduces those of Zn [7,9]. To produce grains with low Cd and high Zn levels, it is therefore necessary to reveal the physiological mechanisms that could differentially regulate the allocation of Cd and Zn to grains.

Relatively less attention has been paid to the phloem transport of Cd and Zn, despite the phloem being the main pathway for the loading of these elements into rice grains [19,20]. Previous studies have shown that the majority (>90%) of Cd and Zn in rice grains is allocated via the phloem after having been exported from phloem source organs or after xylem-to-phloem transfer in the nodes [21,22]. In rice during grain filling, the leaves are the primary phloem source that significantly exports Cd and Zn to the phloem sink, i.e., grains [23,24]. To date, it remains unclear if there are any differences between Cd and Zn in their phloem transport that could be used for differentially regulating the allocation of these elements during grain filling.

It is well accepted that tissue senescence makes the minerals stored in leaves available for further phloem export [25]. During leaf senescence, autophagy catabolizes cellular components gradually [26]. In general, the chloroplast is the first target of autophagic degradation, which turns the leaf from green to yellow [25]. Thus, the minerals intensively contained in the chloroplasts are the first to become available for phloem transport. For example, more than half of nitrogen in leaves is present in Rubisco in chloroplasts, in which phloem export is well synchronized with the onset of leaf senescence [27]. With respect to Zn, the dependency between its phloem export and tissue senescence has long been recognized in cereal crops [28,29]. Indeed, accelerating leaf senescence is suggested as an effective strategy to improve the grain nutrition value for protein, Fe and Zn [30]. By contrast, Cd is relatively less mobile for phloem export from leaves. In rice, Wiggenhauser, et al. [31] revealed that Cd can complex with organic acids in the vacuole or with O-donors in the cell wall, thereby contributing to the immobility of Cd in leaves and stems. These cellular components are degraded or even remain intact at the late stage of autophagy [26], which may lead to the immobility of Cd during senescence. Upon the phloem export from the leaves, the nodes, particularly the uppermost node I, allocate Cd and Zn further to the grain or other organs. Although this allocation process is mainly regulated by OsHMA2, which is shared by Cd and Zn as mentioned above, there are transporters that are highly specific for Cd (OsLCT1) and for Zn (OsZIP3) in regulating the intervascular transfer of these elements [32,33]. When the phloem export of minerals changes during tissue senescence, plants are able to coordinate the expression of nodal transporter genes to meet the sink demand or avoid potential toxicity [34]. Taken together, we hypothesized that the difference between the phloem transport of Cd and Zn relies on their distinct responses to senescence during grain filling.

This study aimed at (1) testing whether Cd and Zn respond differently to senescence in terms of phloem export and subsequent allocation, and (2) understanding the physiological processes that lead to the differences. To this end, the stable isotopes ^111^Cd and ^67^Zn were used to trace the allocation of Cd and Zn from the flag leaf to other parts of rice. Plant senescence was accelerated by applying the phytohormone abscisic acid. The subcellular distribution of Cd and Zn tracers in the flag leaf and the expression of some key transporter genes were also investigated upon tissue senescence.

## 2. Results

### 2.1. Plant Growth and Total Cd and Zn Accumulation

**Figure 1:**
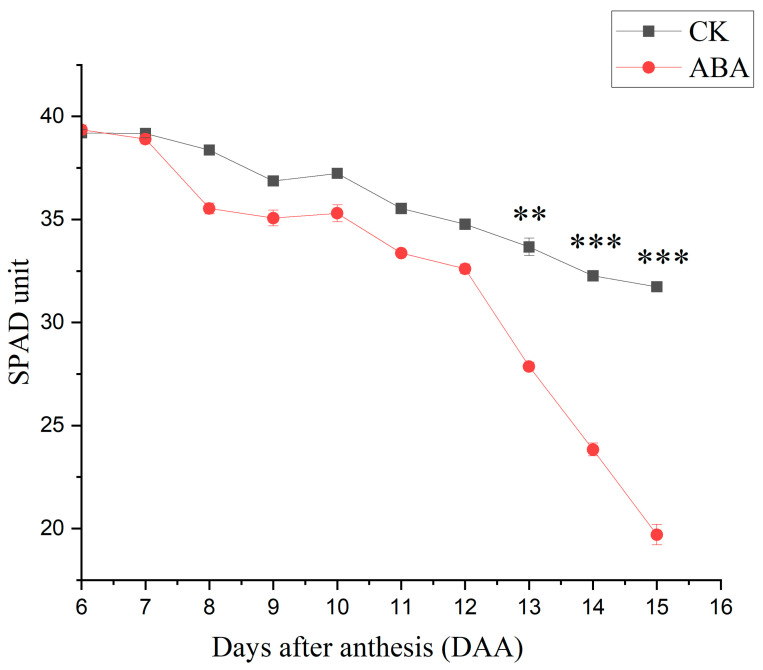
The relative chlorophyll concentration (SPAD units) measured on the flag leaves of the labeled main tillers after hormone treatments. ABA, abscisic acid; CK, blank control. The values are the means of five independent replicates ± standard error. Each replicate represents 20 sampling points randomly distributed on a leaf of one treatment (** *p* < 0.01, or *** *p* < 0.001).

During the entire growth period, the plant grew healthily and no poisoning symptoms of Cd were observed. From the 13th day after anthesis, the relative chlorophyll concentration (SPAD unit) of labeled flag leaves was significantly different between the ABA treatment and the blank control (CK; Figure 1). The SPAD unit decreased by 48% in the ABA plants during the 9 days of hormone treatment, but by only 19% in the CK.

There were no significant differences in the grain yield and plant biomass between ABA and CK treatments (Table 1). The ABA treatment had no impact on the distribution of dry matter among plant organs. Biomass was mostly allocated to the grains in the labeling tiller (including grains, H + R, flag leaves, node-I and stems). For the whole plant, over 75% of biomass was distributed in the shoots (8.81–8.91 g).

In the two treatments, except for the flag leaves labeled with Cd, the total Cd concentration was the highest in the root (Table 2). The total Zn concentration was the highest in node-I, followed by the root. Between 6 and 15 DAA, the total amount of Cd and Zn in grains kept increasing, while no significant differences were observed between the treatments (Table S1). By contrast, compared to the CK, the total amounts of Zn were increased in the node-I by 44% and in the stem by 63% upon the ABA treatment, whereas the amounts of Cd in these organs were not affected. In addition, while the concentrations of Cd were not affected by the ABA, the Zn concentrations were increased by 56% in the other tillers. In addition, in the other tillers and node-I, while the Cd concentrations were not affected by the ABA treatment, the Zn concentrations significantly increased by 56% and 39%, respectively.

After 24 h of isotope-labeled Cd (Cd_lab_) and labeled Zn (Zn_lab_) application, 0.82 μg Cd_lab_ and 1.20 μg Zn_lab_ were absorbed (Table S2).

### 2.2. Phloem Export and Allocation of Foliar Applied Cd and Zn

**Figure 2:**
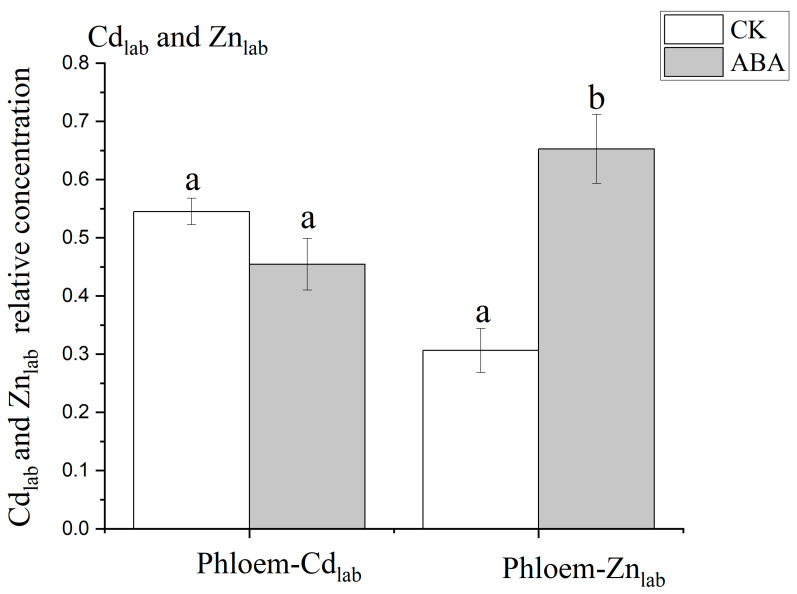
The Cd_lab_ and Zn_lab_ relative concentration in phloem exudates at 15 DAA (after anthesis). ABA, abscisic acid. CK, blank control. Cd_lab_/Zn_lab_, the labeled source of Cd/Zn with an enriched ^111^Cd/^67^Zn isotope. Data are mean values ± standard error (bars in the figures) calculated from four independent replicates. The Cd_lab_/Zn_lab_ concentration in the phloem exudates according to the ratio between the iron concentration and the Cd/Zn concentration in phloem exudates. For each element, different letters within a row indicate significant differences (*p* < 0.05).

In this experiment, the concentration of Cd_lab_ and Zn_lab_ in the phloem exudates was standardized by Fe. The Cd_lab_ concentration in the phloem exudate was similar between the treatments (Figure 2). By contrast, compared to CK, the Zn_lab_ concentration in the phloem exudate increased by 110% under ABA treatment (*p* < 0.05).

**Figure 3:**
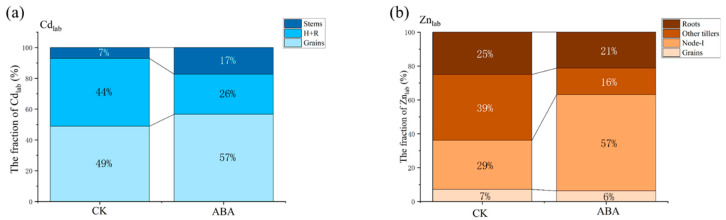
The fraction of Cd_lab_ (**a**) and Zn_lab_ (**b**) exported from the labeled flag leaf to the other organs in the labeling tiller at 15 DAA (after anthesis). ABA, abscisic acid. CK, blank control. Cd_lab_/Zn_lab_, the labeled source of Cd/Zn with an enriched ^111^Cd/^67^Zn isotope. The data are the mean values calculated from four independent replicates.

As shown in Table 3 and Table S3, there were no significant differences between the treatments in the amount of Cd_lab_ (0.10–0.12 μg) and Zn_lab_ (0.81–0.82 μg) remobilized from the flag leaves. The Zn_lab_ exported from flag leaves was distributed into various parts of the rice. The Zn_lab_ concentrations were 0.6–0.8 μg kg^−1^ in the rhizosphere soil where, however, no Cd_lab_ was detected. At maturity, the exported Zn_lab_ was mainly found in the grains, stems, node-I, other tillers and roots, whereas Cd_lab_ was found only in the grains, H + R and stems (Figure 3). In the ABA treatment, the amount of Zn_lab_ in node-I was significantly higher than that that in the CK (Table 3). In the ABA treatment, 57% of the exported Zn_lab_ was allocated to the node-I was significantly higher than the fraction (29%) in the CK (Figure 3b).

### 2.3. Subcellular Distribution of Foliar-Applied Cd and Zn

**Figure 4:**
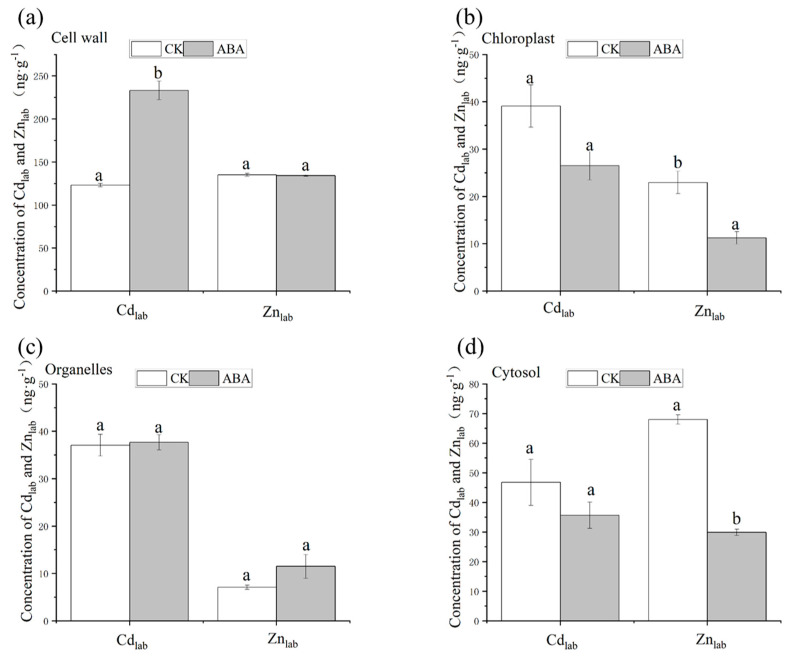
The concentrations of Cd_lab_ and Zn_lab_ in flag leaves subcellular fractions at 15 DAA (after anthesis). ABA, abscisic acid. CK, blank control. Cd_lab_/Zn_lab_, the labeled source of Cd/Zn with an enriched ^111^Cd/^67^Zn isotope. (**a**) The concentrations of Cd_lab_ and Zn_lab_ in cell wall. (**b**) The concentrations of Cd_lab_ and Zn_lab_ in chloroolast. (**c**) The concentrations of Cd_lab_ and Zn_lab_ in organelles. (**d**) The concentrations of Cd_lab_ and Zn_lab_ in cytosol. The data are mean values ± standard error (bars in the figures) calculated from three independent replicates. For each element, the different letters within a row indicate significant differences (*p* < 0.05).

The ABA treatment significantly affected the concentrations of Cd_lab_ and Zn_lab_ in the subcellular fractions extracted from the flag leaves (Figure 4). In the cell wall fraction, the concentration of Cd_lab_ increased by 89% but that of Zn_lab_ remained stable with ABA application compared to CK. Regardless of the treatment, more than 50% of Cd_lab_ was sequestrated in the cell wall (Figure S1). By contrast, in the chloroplast and cytosol fractions, ABA treatment significantly lowered the concentration of Zn_lab_ by more than 50% but had no impact on the level of Cd_lab_. In the organelle fraction, for both Cd_lab_ and Zn_lab_, the concentrations were similar between the treatments.

### 2.4. Expression Profile of OsHMA2, OsLCT1 and OsZIP3 in the Flag Leaves and Node-I

**Figure 5:**
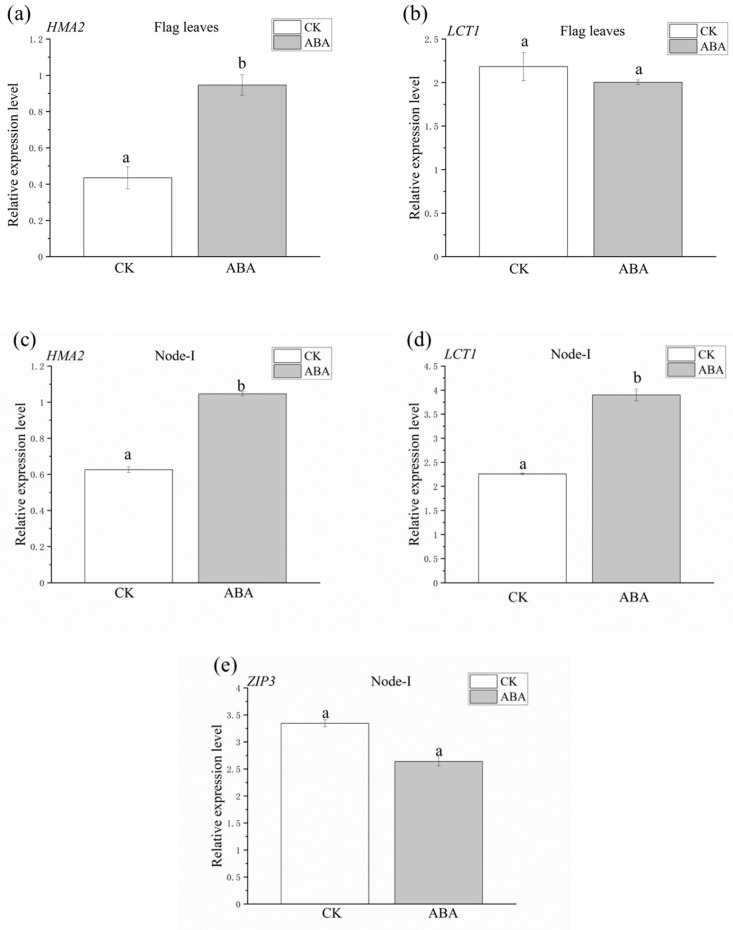
The relative expression patterns of *OsHMA2*, *OsLCT1* and *OsZIP3* in the rice flag leaves and the node-I by qRT-PCR analysis at 15 DAA (after anthesis). ABA, abscisic acid. CK, blank control. (**a**) The relative expression patterns of *OsHMA2* in flag leaves. (**b**) The relative expression patterns of *OsLCT1* in flag leaves. (**c**) The relative expression patterns of *OsHMA2* in node-I. (**d**) The relative expression patterns of *OsLCT1* in node-I. (**e**) The relative expression patterns of *OsZIP3* in node-I. The data are mean values ± standard error (bars in the figures) calculated from three independent replicates. For each element, the different letters within a row indicate significant differences (*p* < 0.05).

In the flag leaves, the ABA treatment significantly increased the relative expression level of *OsHMA2* by 2.2 times compared to the CK, but had no impact on the expression of *OsLCT1* (Figure 5a,b). In the node-I, both the expression levels of *OsHMA2* and *OsLCT1* were promoted under the ABA treatment by around two times (Figure 5c,d). The relative expression level of *OsZIP3* remained stable regardless of the treatments (Figure 5e).

## 3. Material and Methods

### 3.1. Plant Culture

Rice (*Oryza sativa* L.) cultivar ‘Meixiangzhan 2′ was used in this study, which has a higher risk of accumulating Cd in the grain to levels exceeding the regulatory limit (Li Guijie, 2016). Seeds were surface sterilized by using 2% NaClO solution for 20 min and rinsed five times with deionized water. The seeds were germinated in the dark for 5 days at 30 °C. After germination, the seedlings were initially grown in hydroponics for 4 weeks, with 15 h of light at 25 °C, and 9 h of darkness at 18 °C. The humidity was set to 60%–65% and the light intensity was set to 25 klx. The nutrient solution was a modified Hoagland solution with the same composition as described in Yan, et al. [35]: 0.5 mM MgSO_4_, 1.25 mM Ca(NO_3_)_2_, 1.25 mM KNO_3_, 0.25 mM KH_2_PO_4_, 46.25 μM H_3_BO_3_, 1.0 μM MnSO_4_, 10 μM ZnSO_4_, 2.0 μM CuSO_4_, 75 μM KCl, 1.0 μM (NH_4_)_6_Mo_7_O_24_, 100 μM Na_2_FeEDTA, and 100 nM CdCl_2_. Besides, 29.6 μM HEDTA and 1.5 mM MES buffer were added. The pH was adjusted to 5.6 with solid KOH. The nutrient solution was replaced every 1–3 day(s).

The seedlings were transplanted at the tillering stage (33 days after germination) to pots (155 mm in height and 162 mm in diameter; one plant per pot) filled with substrate mixing three volumes of peat substrate (Fine, Jiffy, Zwijndrecht, Holland) and one volume of medium grain quartz sand (particle size 0.5–1 mm). Slow-release fertilizer Plantacote Depote 6M (NPK 14-9-15, Meyer, Rellingen, Germany) was supplied to the substrate at the rate of 1 g per liter, and 0.1 g per liter micronutrient fertilizer Fetrilon-Combi (0.7% Mg, 1.5% B, 0.5% Cu, 4% Fe, 3% Mn, 0.05% Mo, 4% Zn, COMPO EXPERT GmbH, Poznan, Poland) was supplied. The rice plants were planted in an outdoor plastic greenhouse from April to early July 2022. The rice was exposed to natural sunlight and ventilation in Guangzhou (23°26′ N, 113°23′ E). The daily average temperature was 26.8–31.0 °C (min 15 °C, max 38 °C) during cultivation. The monthly average relative humidity was 75%–80% (data: Chinese National Meteorological Centre). The rice was re-flooded every dusk with tap water.

The phenology was carefully observed for each plant. We defined ‘anthesis’ when the anthers were visible, and the tiller carrying the first anthesis floret was defined as the main tiller [36]. When 3 florets carried anthers, these 3 tillers were kept while other tillers were removed by cutting.

### 3.2. Stable Cadmium and Zinc Isotope Labeling

The ^111^Cd-enriched isotope and ^67^Zn-enriched isotope were purchased from Isoflex (the United States). The isotopic abundances of Cd and Zn are shown in Table S4. A solution containing 1.0 mM ^111^Cd and 2.0 mM ^67^Zn was prepared and adjusted to neutral (pH = 6.5) using MES and KOH. To enhance the surface adhesion of solutions toward leaves, Tween 80 (0.05% *v*/*v*) was added. At 5 days after anthesis (DAA), ten 5 μL droplets of the isotope solution were applied to the flag leaves of main tillers. Note that the other 2 tillers were not labeled. This time was selected considering that grain accumulates dry matter rapidly between 4 and 18 DAA [37,38]. After 24 h, the treated flag leaves were rinsed with a solution containing 2% nitric acid (HNO_3_) and 3% ethanol to remove the unabsorbed isotope label [39,40].

### 3.3. Plant Hormone Treatment

Two treatments were established with 10 replicates each. Abscisic acid (ABA) was used to accelerate the plant senescence [41]. ABA solution was prepared by dissolving dextrorotatory ABA powder in NaOH and diluted to 200 μM with ultrapure water. Meanwhile, a solution with the same concentration of NaOH was prepared as the blank control (CK). These solutions also contained Tween 80 (0.05% *v*/*v*), which can reduce the surface tension of rice leaves effectively, and prolong the residence time of hormones on the leaf surface. For all replicates of each treatment, in total 250 mL of the solution was sprayed on the plants from the top side every 24 h, which lasted 9 days. Before the hormone treatment, the chlorophyll relative concentration of each plant was measured using a soil and plant analyzer development (SPAD-502, Minolta, Milton Keynes, UK). The flag leaves of the plants were taken as the measurement objects, and 20 points were randomly measured and averaged, each group was measured five times and recorded. The hormone treatment was continued for 9 days, until the SPAD values were sufficiently distinguished between the treatments.

### 3.4. Phloem Exudate Extraction

Flag leaves were collected freshly after the hormone treatment (at 15 DAA) for phloem exudate extraction, according to Tetyuk, et al. [42]. Briefly, for each treatment, 4 pieces of fresh flag leaves (including the blade and sheath) from the main tillers of different plants subjected to the same treatment were cut at the node level and recut (about 1 mm) at the sheath bottom in a petri dish containing 20 mM K_2_-EDTA solution. Then, the leaves were quickly transferred to a centrifuge tube (5 mL, Eppendorf, Hamburg, Germany) containing 1.4 mL of 20 mM K_2_-EDTA. After immersing the sheath bottom in the solution, the leaves were placed in a dark and humid environment for 1 h. After bath washing the sheath bottom using flowing ultrapure water, the flag leaves were transferred to a new tube containing 1.4 mL ultrapure water. The phloem exudate was extracted to the water for 5 h in a dark and humid environment. Deng, et al. [43] suggested that Fe can be used as a reference element to standardize the variations in the extraction efficiency as the concentration of Fe is relative constant in the phloem exudates. Therefore, in the present study, the concentrations of Cd_lab_ and Zn_lab_ in the phloem exudates were standardized and expressed as the ratios between Zn/Cd and Fe concentrations.

### 3.5. Extraction of Subcellular Fractions

From the extras’ replicates, the flag leaves were collected freshly for the extraction of subcellular fractions, according to the method used in Wu, et al. [44]. Briefly, each fresh flag leaf was homogenized with 10.0 mL of precooled (4 °C) extraction solution containing 250 mmol·L^−1^ sucrose, 50 mmol·L^−1^ Tris HCl (pH = 7.50) and 1 mmol·L^−1^ dithiocarbaryl. The homogenate was filtered by a 240 μM nylon cloth, the rest in the nylon cloth was the cell wall fraction that may contain the cell walls and cell wall debris. After that, the filtrate was collected into a 50 mL centrifuge tube, centrifuged at 1500× *g* with a high-speed freezing centrifuge for 10 min, and the precipitation was the chloroplast fraction. The supernatant was further centrifuged at 15,000× *g* for 30 min. The precipitation was the organelle fraction that may contain the cell membrane and organelles (apart from the chloroplast). The supernatant was the cytosol fraction that may contain the cytoplasm and vacuoles. All steps were performed at 4 °C.

### 3.6. Plant Harvesting

To reveal the allocation of Cd and Zn upon the hormone treatments, the plants were harvested at 6 DAA (after anthesis) and at 15 DAA (after anthesis). The roots were carefully separated from the soil with a tap-water flow and deionized water, then bathed in 4 L of 1 μM DTPA solution for 10 min and rinsed with deionized water for 1–2 min to remove the Cd adsorbed onto the apoplast [45]. For the shoots, the labeling tillers were divided into grains, husk + rachis (H + R), flag leaves, node-I (the uppermost node), stems (including the lower nodes and internodes), and the lower leaves, while the other tillers were mixed as one sample. All these shoot organs were washed with tap water followed by deionized water. Then, all the plant samples were dried to constant weight in an oven at 70 °C, weighed, and ground with tungsten carbide cups and balls. In addition, rhizosphere soil was collected within 2 cm surrounding the rice root [46], air-dried, and sieved to <80 μm.

### 3.7. Sample Processing

The samples of phloem exudate were diluted to 5 mL with deionized water, and 100 μL concentrated HNO_3_ was added. The phloem exudate samples were determined for the total concentration of Zn, Fe and Cd, and for the isotope ratio of ^111^Cd/^110^Cd and ^67^Zn/^66^Zn.

The subcellular fractions samples were digested with 5 mL nitric acid by an electric heating plate (JinRongYuan, JRY-D350-D, Changsha, China) at 105 °C for 180 min and dilute to 20 mL to determine the total concentration of Zn and Cd, and the isotope ratio of ^111^Cd/^110^Cd and ^67^Zn/^66^Zn.

About 0.10 g of the plant samples and soil samples were preliminarily digested for >12 h in 1 mL 69% HNO_3_ and 3 mL ultrapure water. After preliminary digestion, 1 mL 30% (*w*/*v*) H_2_O_2_ was added. The samples were digested by a microwave digestion extraction system (CEM, MARS6, Boston, MA, USA) at 120 °C for 10 min, 180 °C for 10 min and 210 °C for 20 min. The digests were diluted to 20 mL using ultrapure water and filtrated through water phase needle filters (0.45 μm).

The total Cd concentration was measured by atomic absorption spectroscopy (AAS, JENA, Contro AA800, Jena, Germany). The total Zn and Fe concentrations were measured by inductively coupled plasma optical emission spectrometer (ICP-OES, PerkinElmer, Avio 500, Waltham, MA, USA). The isotope ratios of ^111^Cd/^110^Cd and ^67^Zn/^66^Zn were measured by inductively coupled plasma mass spectrometry (ICP-MS, PerkinElmer, NexIon 350D, Waltham, MA, USA).

### 3.8. RNA Extraction and qRT-PCR Analysis

The RNA extraction and qRT-PCR analysis were carried out according to Sun, et al. [47]. Total RNA was extracted from fresh plant node-I and flag leaves using the FastPure Plant Total RNA Isolation Kit (Vazyme, Nanjing, China) and then reverse-transcribed using the HiScript III RT SuperMix for qPCR (Vazyme, Nanjing, China). qRT-PCR was performed using the StepOne Real-Time PCR System (StepOne, Shanghai, China). The relative expression levels were computed by the 2^−ΔΔCT^ method of relative quantification. *Oryza sativa* L. actin gene (*OsActin*) was used as the internal control, with primers being shown in Table S5.

### 3.9. Data Analysis

For each organ of each plant, the amount of total Cd ($Q_{Cd}^{tot}$ [μg]) and the amount of total Zn ($Q_{Zn}^{tot}$ [μg]) was calculated by multiplying the dry weight (DW [g]) of the organ by the concentrations of total Cd and total Zn ($C_{Cd}^{tot}$ [μg] and $C_{Zn}^{tot}$ [μg]), respectively:


(1) $$Q_{\mathrm{Cd}}^{\mathrm{tot}}=\mathrm{DW}\cdot C_{\mathrm{Cd}}^{\mathrm{tot}}$$

(2) $$Q_{\mathrm{Zn}}^{\mathrm{tot}}=\mathrm{DW}\cdot C_{\mathrm{Zn}}^{\mathrm{tot}}$$

There are two sources of Cd/Zn in the samples: the labeled source of Cd/Zn with an enriched ^111^Cd/^67^Zn isotope (Cd_lab_/Zn_lab_) and the unlabeled source of Cd/Zn with a natural Cd/Zn isotopic composition (Cd_nat_/Zn_nat_). The sum of these Cd/Zn sources is denoted as total Cd/Zn.

For the phloem exudate samples, each plant sample and the subcellular fraction samples, the fraction of Cd_lab_ (FCd_lab_ [%]) and Zn_lab_ (FZn_lab_ [%]) in the organ was calculated from the ^111^Cd/^110^Cd ratio and ^67^Zn/^66^Zn ratio determined in the corresponding digest:

(3) FCdlab=ACd110nat·Cd111Cd110-ACd111natA111Cdlab-A111Cdnat-Cd111Cd110·(ACd110lab-ACd110nat)

(4) FZnlab=AZn66nat·Zn67Zn66-AZn67natAZn67lab-A67Znnat-Zn67Zn66·(A66Znlab-A66Znnat)

where A refers to the abundance of the isotope provided in Table S4.

The amount of Cd_lab_ (${QCd}_{lab}\left[\mu g\right]$) and Zn_lab_ (${QZn}_{lab}\left[\mu g\right]$) in each organ was calculated by multiplying the total amount by FCd_lab_ or FZn_lab_:


(5) $${QZn}_{lab}={FZn}_{lab}\cdot Q_{Zn}^{tot}$$

(6) $${QCd}_{lab}={FCd}_{lab}\cdot Q_{Cd}^{tot}$$

For Cd_lab_ and Zn_lab_ the net amount exported ($Q_{Cd}^{exp}$ and $Q_{Zn}^{exp}$) from the labeled flag leaf was estimated as the decreased amount of Cd_lab_/Zn_lab_ in the flag leaf before and after the hormone treatment (i.e., between 6 and 15 DAA), given that the decrease was statistically significant.

Statistical analysis with Student’s *t*-test was used to identify the significant differences between the treatments. Statistical analyses were conducted using Excel 2016 (Microsoft, Washington, DC, USA) for Windows, and all graphs were drawn by OriginPro 2023 (OriginLab, Northampton, MA, USA).

## 4. Discussion

### 4.1. ABA-Induced Senescence Fortified the Zn Phloem Export, but Had No Impact on the Cd Phloem Export

Compared to CK, the significantly decreased SPAD value showed that ABA significantly accelerated the flag leaves’ senescence (Figure 1). Previous studies showed that senescence promotes the remobilization of minerals from leaves in crops [33,48,49]. Phloem is the only pathway of Zn and Cd remobilized from leaves [22,50]. In the present study, the ABA treatment markedly enhanced the phloem export of Zn_lab_ applied to the flag leaves, but had no impact on the phloem export of Cd_lab_ (Figure 2). The changes in the subcellular distribution of Cd and Zn upon ABA-induced senescence were investigated to understand the physiological mechanism behind this observation.

It is generally proposed that Zn is mainly present in the cytosol and functional organelles (e.g., chloroplast and mitochondrion), given its essentiality for enzyme functioning, while the potentially toxic Cd is mainly immobilized by the cell wall or compartmentalized in the vacuole [3,51]. ABA-induced senescence promoted the release of Zn_lab_ in chloroplasts and cytosol components significantly. The total proportion of Zn_lab_ allocated to these components in the CK was 64%, while ABA-induced senescence resulted in a large outflow of Zn_lab_ from both of them (Figure 4) and a significant decrease in the proportion (down to 22%; Figure S1). Consequently, the phloem export of Zn_lab_ markedly increased (Figure 2). By contrast, ABA-induced senescence promoted the distribution and fixation of Cd_lab_ in flag leaves to the cell wall component, which may adsorb some, if not all, Cd_lab_ released by the other cellular components during senescence, thus limiting the phloem export of Cd_lab_. The ABA treatment promoted the release of Cd from some subcellular components, as the concentrations of Cd_lab_ in the chloroplast and cytosol components decreased (but the significance may be diluted by variability among replicates) (Figure 4b,d). In the flag leaves, Cd_lab_ was mainly stored in the cell wall component (Figure 4a), which was similar to many other studies [52,53]. In addition, cell wall precipitation is a defense mechanism of plant cells against heavy metal toxicity [54]. During grain filling, with the senescence of rice, the Cd storage as Cd–O in the cell wall may contribute to the immobilization of Cd in the flag leaves [31]. The remobilization of Zn in flag leaves may depend on the plant’s developmental stage [55]. Similar transportation of Zn and Cd in the phloem has been shown by Page and Feller [56]. It has been found that Cd in the older leaves was subsequently remobilized in the phloem only at trace levels compared to Zn [56]. This study suggests that ABA-induced senescence did not promote the release of Cd from leaf cells, which may explain the limited phloem export of Cd. Therefore, compared to Cd, Zn appeared to be more mobile in the flag leaves during ABA-induced senescence, which may explain the differential effect of senescence on the phloem export of Cd and Zn from the flag leaves.

However, the enhanced Zn_lab_ phloem export did not result in more Zn_lab_ export from the flag leaves (Table 3). This may be due to a balanced import and export of Zn_lab_ mass in the flag leaves. In this study, we found that Zn_lab_ applied to the flag leaf was allocated to the roots, which was similar to Haslett [57] reported in wheat. Meanwhile, according to Table S1, compared with CK, the root-to-shoot ratio of total Zn amount increased significantly in the ABA treatment, indicating that more Zn_lab_ was translocated from the root, which may result in the absence of apparent Zn_lab_ export in the flag leaves. Therefore, the mass balance data cannot distinguish between the leaf source and root source, but the Zn_lab_ in the phloem sap can be determined to be exported from the leaves.

### 4.2. ABA-Induced Senescence Affects the Allocation of Phloem-Exported Zn by Altering the Expression of Transporter Genes in Flag Leaves and Nodes

In accordance with previous studies, Cd and Zn were found to be highly enriched in the uppermost Node-I compared to other organs [21,58,59]. The node-I is known as a key hub for the allocation of Cd and Zn in rice. In addition, the flag leaves were the phloem sources of Cd and Zn in the present study. Given these, in the node-I and flag leaves, we investigated the effect of ABA-induced senescence on the relative expression levels of some key transporter genes (*OsLCT1*, *OsHMA2* and *OsZIP3*).

*OsLCT1* is known to be mainly expressed in leaf blades and nodes and facilitates the export of Cd, but not Zn [13,32]. The expression level for *OsLCT1* in the flag leaves was not affected by ABA-induced senescence (Figure 5b), which corresponded to the observation that senescence had no effect on Cd export from the flag leaves found in our study (Figure 2). ABA-induced senescence promoted the expression of *OsLCT1* in the node-I (Figure 5d), but had no effect on the distribution of Cd in node-I (Table 3). It was reported that decreased expression of *OsLCT1* can lead to a decrease in the concentration of Cd in the phloem, and *OsLCT1* in the diffuse vascular bundles of node-I is important for Cd transport into grains [32]. We found that senescence did not promote the expression of *OsLCT1* in flag leaves (Figure 5b). This result may explain why ABA-induced senescence had no effect on the phloem export of Cd_lab_ from the flag leaves and the allocation of Cd_lab_ through the phloem toward the other organs (e.g., node-I, grains).

Although ABA-induced senescence promoted the export of Zn in the phloem, we did not find a significant increase in Zn in the grains. This is mainly due to the significant accumulation of Zn in the node-I in the ABA treatment (Table 3). *OsHMA2* in flag leaves was strongly upregulated by ABA-induced senescence (Figure 5a). *OsHMA2* has been proven to play a key role in the phloem transport of Zn and Cd, expressed mainly in node-I at the flowering stage, and also expressed in flag leaves [13]. OsHMA2 is localized at the enlarged and diffuse vascular bundles in the nodes and regulates the preferential distribution of Cd and Zn through the phloem to the developing tissues [18]. It was found that the root-to-shoot translocation of Zn via OsHMA2 remained stable when the concentration of Cd in the nutrient solution increased from 0 to 4 nM, indicating that Cd can hardly compete with Zn for OsHMA2 at far lower concentrations than Zn [60]. In this study, the concentration of total Zn in flag leaves was much higher than that of total Cd (The ratio of total Zn to total Cd concentration was greater than 10). Therefore, compared to Cd, Zn was more competitive for the binding site of OsHMA2, which may mainly promote the transport of Zn in the phloem.

ABA-induced senescence had no effect on the expression level of *OsZIP3* in the node-I (Figure 5e). Sasaki, et al. [33] found that OsZIP3 is localized in rice nodes and is an essential transporter responsible for the distribution of Zn to developing tissues through unloading Zn from the xylem. In this study, while ABA treatment significantly promoted the root-to-shoot translocation of Zn, it did not increase the level of zinc in grains (Table S1). This might be due to the unaffected expression level of *ZIP3* in node-I, limiting the unloading Zn from the xylem the subsequent allocation of Zn toward grains.

## 5. Conclusions

Senescence induced by abscisic acid (ABA) promoted the phloem export of Zn from flag leaves, but not Cd, because senescence caused significant Zn release from the chloroplast and cytosol fractions of leaf cells, while Cd was immobilized in the cell wall fractions. Meanwhile, the expression of *OsHMA2* in the flag leaves was promoted by senescence, which facilitated the loading of Zn into the phloem. Nevertheless, due to the strong sequestration of phloem-exported Zn in the uppermost node-I, the accumulation of Zn in the grain was not affected by accelerated senescence. By contrast, although senescence enhanced *OsLCT1* expression in node-I, the allocation of phloem-exported Cd was also not different in the node-I thanks to the limiting *OsLCT1* expression of flag leaves upon ABA treatment. These results showed that agronomic measures, such as irrigation and fertilization, could be used to differentially regulate the allocation of Zn and Cd in rice during grain filling through their effects on plant senescence. To effectively manage the grain Zn and Cd levels, however, further studies are still required to better understand the response of node-I in distributing the phloem-exported Zn and Cd toward plant organs.

## Figures and Tables

**Table 1:** The dry weight of each organ in the main tiller and the dry weight of other tillers, shoot and the whole plan in rice subjected to CK and ABA treatments harvested at 15 days after anthesis. Values are means ± standard error of four independent replicates.

	Dry Weight	
g		
Treatment	CK	ABA
Grains	1.15 ± 0.25 a	0.97 ± 0.38 a
H + R	0.52 ± 0.08 a	0.62 ± 0.09 a
Flag leaves	0.37 ± 0.05 a	0.31 ± 0.04 a
Node-I	0.03 ± 0.00 a	0.03 ± 0.00 a
Stems	0.51 ± 0.07 a	0.60 ± 0.10 a
Lower leaves	0.90 ± 0.19 a	0.80 ± 0.09 a
Other tillers	5.44 ± 0.09 a	5.49 ± 0.46 a
Roots	2.68 ± 0.32 a	2.40 ± 0.15 a
Labeling tiller	3.47 ± 0.56 a	3.32 ± 0.24 a
Straw	10.44 ± 0.72 a	10.24 ± 0.32 a
Shoots	8.91 ± 0.64 a	8.81 ± 0.43 a
Whole plant	11.59 ± 0.95 a	11.21 ± 0.49 a

The whole plant was the sum of the dry weight of each organ. Shoot, the total aboveground biomass. Labeling tiller, the total aboveground biomass of labeling tiller. Straw, the total amount of aboveground parts except grains. H + R, husk + rachis. The letter ‘a’ indicates the corresponding organ between two treatments had no significant differences (*p* > 0.05).

**Table 2:** The concentrations of total Cd and Zn in each organ in the labeling tiller harvested at 6 days after anthesis (DAA) and at 15 DAA (after anthesis) in rice. Values are means ± standard error of four independent replicates.

	Cd Concentration (μg Cd g^−1^ DW)			Zn Concentration (μg Zn g^−1^ DW)		
Harvest	6 DAA	15 DAA		6 DAA	15 DAA	
	6 DAA	CK	ABA	6 DAA	CK	ABA
Grains	0.04 ± 0.03	0.12 ± 0.01 a	0.14 ± 0.02 a	22.6 ± 1.6	29.8 ± 3.4 a	32.6 ± 1.9 a
H + R	0.15 ± 0.06	0.17 ± 0.04 a	0.19 ± 0.05 a	47.4 ± 2.7	30.5 ± 4.2 a	34.6 ± 4.5 a
Flag leaves	3.35 ± 0.08	4.62 ± 0.60 a	3.28 ± 0.27 a	46.7 ± 5.8	30.6 ± 2.6 a	42.8 ± 7.0 a
Node-I	0.13 ± 0.01	0.72 ± 0.14 a	0.31 ± 0.07 a	304.4 ± 28.5	393.5 ± 24.2 a	605.4 ± 20.1 b
Stems	0.05 ± 0.00	0.17 ± 0.03 a	0.12 ± 0.04 a	82.4 ± 8.2	97.7 ± 1.2 a	133.5 ± 6.1 a
Lower leaves	0.15 ± 0.03	0.28 ± 0.06 a	0.41 ± 0.09 a	45.4 ± 0.5	32.4 ± 3.5 a	48.6 ± 10.1 a
Other tillers	0.10 ± 0.03	0.13 ± 0.05 a	0.22 ± 0.04 a	49.0 ± 0.6	39.3 ± 1.1 a	61.0 ± 5.3 b
Roots	0.89 ± 0.25	1.22 ± 0.19 a	0.76 ± 0.14 a	219.5 ± 63.2	294.9 ± 73.0 a	218.4 ± 63.8 a

H + R, husk + rachis. For each element at 15 DAA, the different letters within a row indicate significant differences between two treatments (*p* < 0.05).

**Table 3:** The net changes of Cd_lab_ and Zn_lab_ between 6 DAA (after anthesis) and at 15 DAA (after anthesis) in the rice in CK and ABA treatments. Values are means ± standard error of four independent replicates.

Net Changes of Cd_lab_ (μg Cd_lab_ Plant^−1^)		
	CK	ABA
Grains	0.06 ± 0.01 a	0.05 ± 0.01 a
H + R	0.05 ± 0.03 a	0.03 ± 0.02 a
Node-I	ns	ns
Stems	0.01 ± 0.00 a	0.02 ± 0.01 a
Lower leaves	n.d	n.d
Other tillers	n.d	n.d
Roots	n.d	n.d
Flag leaves	−(0.12 ± 0.02) a	−(0.10 ± 0.05) a
**Net changes of Zn_lab_ (μg Zn_lab_ plant^−1^)**		
	**CK**	**ABA**
Grains	0.03 ± 0.01 a	0.04 ± 0.01 a
H + R	ns	ns
Node-I	0.12 ± 0.03 b	0.33 ± 0.04 a
Stems	ns	ns
Lower leaves	ns	ns
Other tillers	0.16 ± 0.05 a	0.09 ± 0.04 a
Roots	0.10 ± 0.05 a	0.12 ± 0.07 a
Flag leaves	−(0.82 ± 0.03) a	−(0.81 ± 0.03) a

H + R, husk + rachis. n.d. Cd_lab_ and Zn_lab_ under the detection limit at 15 DAA (after anthesis). Cd_lab_/Zn_lab_, the labeled source of Cd/Zn with an enriched ^111^Cd/^67^Zn isotope. ns stands for no significant difference. H + R, husk + rachis. For each element, the different letters within a row indicate significant differences in the corresponding organs between CK and ABA (*p* < 0.05).

## Data Availability

No new data were created or analyzed in this study. Data sharing is not applicable to this article.

## Supplementary Materials

The following supporting information can be downloaded at: https://www.mdpi.com/article/10.3390/plants12091902/s1, Table S1: Amount of total Cd and Zn in each organ harvested at 6 DAA (after anthesis) and at 15 DAA (after anthesis) in rice; Table S2: Cd_lab_ and Zn_lab_ absorption in flag leaves during 24 h; Table S3: The amount Cd_lab_ and Zn_lab_ among the rice in two treatments at 6 DAA (after anthesis) and at 15 DAA (after anthesis); Table S4: isotopic abundances of Zn and Cd (data from the isotope certificate); Table S5: Primers for qRT-PCR. Figure S1: Subcellular distribution proportion of Cdlab (a) and Znlab (b) in flag leaves of rice at 15DAA (after anthesis).

## References

[1] Clemens S. (2019). Safer food through plant science: Reducing toxic element accumulation in crops. J. Exp. Bot..

[2] Cakmak I., Kutman U.B. (2017). Agronomic biofortification of cereals with zinc: A review. Eur. J. Soil Sci..

[3] Clemens S. (2022). The cell biology of zinc. J. Exp. Bot..

[4] Järup L., Åkesson A. (2009). Current status of cadmium as an environmental health problem. Toxicol. Appl. Pharmacol..

[5] Timmer C.P. (2014). Food Security in Asia and the Pacific: The Rapidly Changing Role of Rice. Asia Pac. Policy Stud..

[6] Hu Y., Cheng H., Tao S. (2016). The challenges and solutions for cadmium-contaminated rice in China: A critical review. Environ. Int..

[7] Smolders E., Wagner S., Prohaska T., Irrgeher J., Santner J. (2020). Sub-millimeter distribution of labile trace element fluxes in the rhizosphere explains differential effects of soil liming on cadmium and zinc uptake in maize. Sci. Total Environ..

[8] Hussain S., Khan A.M., Rengel Z. (2019). Zinc-biofortified wheat accumulates more cadmium in grains than standard wheat when grown on cadmium-contaminated soil regardless of soil and foliar zinc application. Sci. Total Environ..

[9] Chen H., Zhang W., Yang X., Wang P., McGrath S.P., Zhao F.-J. (2018). Effective methods to reduce cadmium accumulation in rice grain. Chemosphere.

[10] Chaney R.L. (2010). Cadmium and Zinc. Trace Elements in Soils.

[11] Yan B.-F., Dürr-Auster T., Frossard E., Wiggenhauser M. (2021). The Use of Stable Zinc Isotope Soil Labeling to Assess the Contribution of Complex Organic Fertilizers to the Zinc Nutrition of Ryegrass. Front. Plant Sci..

[12] Lee S., An G. (2009). Over-expression of OsIRT1 leads to increased iron and zinc accumulations in rice. Plant Cell Environ..

[13] Ma J.F., Shen R.F., Shao J.F. (2021). Transport of cadmium from soil to grain in cereal crops: A review. Pedosphere.

[14] Ueno D., Iwashita T., Zhao F.-J., Ma J.F. (2008). Characterization of Cd translocation and identification of the Cd form in xylem sap of the Cd-hyperaccumulator *Arabidopsis halleri*. Plant Cell Physiol..

[15] Kawakami Y., Bhullar N.K. (2018). Molecular processes in iron and zinc homeostasis and their modulation for biofortification in rice. J. Integr. Plant Biol..

[16] Stomph T.J., Jiang W., Van Der Putten P.E., Struik P.C. (2014). Zinc allocation and re-allocation in rice. Front. Plant Sci..

[17] Kashiwagi T., Shindoh K., Hirotsu N., Ishimaru K. (2009). Evidence for separate translocation pathways in determining cadmium accumulation in grain and aerial plant parts in rice. BMC Plant Biol..

[18] Yamaji N., Xia J., Mitani-Ueno N., Yokosho K., Feng Ma J. (2013). Preferential delivery of zinc to developing tissues in rice is mediated by P-type heavy metal ATPase OsHMA2. Plant Physiol..

[19] Yoneyama T., Gosho T., Kato M., Goto S., Hayashi H. (2010). Xylem and phloem transport of Cd, Zn and Fe into the grains of rice plants (*Oryza sativa* L.) grown in continuously flooded Cd-contaminated soil. Soil Sci. Plant Nutr..

[20] Uraguchi S., Fujiwara T. (2013). Rice breaks ground for cadmium-free cereals. Curr. Opin. Plant Biol..

[21] Nishiyama R., Tanoi K., Yanagisawa S., Yoneyama T. (2013). Quantification of zinc transport via the phloem to the grain in rice plants (*Oryza sativa* L.) at early grain-filling by a combination of mathematical modeling and 65Zn tracing. Soil Sci. Plant Nutr..

[22] Tanaka K., Fujimaki S., Fujiwara T., Yoneyama T., Hayashi H. (2007). Quantitative estimation of the contribution of the phloem in cadmium transport to grains in rice plants (*Oryza sativa* L.). Soil Sci. Plant Nutr..

[23] Wu C.Y., Lu L.L., Yang X.E., Feng Y., Wei Y.Y., Hao H.L., Stoffella P.J., He Z.L. (2010). Uptake, translocation, and remobilization of zinc absorbed at different growth stages by rice genotypes of different Zn densities. J. Agric. Food Chem..

[24] Zhou H., Zhu W., Yang W.-T., Gu J.-F., Gao Z.-X., Chen L.-W., Du W.-Q., Zhang P., Peng P.-Q., Liao B.-H. (2018). Cadmium uptake, accumulation, and remobilization in iron plaque and rice tissues at different growth stages. Ecotoxicol. Environ. Saf..

[25] Distelfeld A., Avni R., Fischer A.M. (2014). Senescence, nutrient remobilization, and yield in wheat and barley. J. Exp. Bot..

[26] Pottier M., Masclaux-Daubresse C., Yoshimoto K., Thomine S. (2014). Autophagy as a possible mechanism for micronutrient remobilization from leaves to seeds. Front. Plant Sci..

[27] Masclaux C., Quilleré I., Gallais A., Hirel B. (2001). The challenge of remobilisation in plant nitrogen economy. A survey of physio-agronomic and molecular approaches. Ann. Appl. Biol..

[28] Maillard A., Etienne P., Diquelou S., Trouverie J., Billard V., Yvin J.C., Ourry A. (2016). Nutrient deficiencies in *Brassica napus* modify the ionomic composition of plant tissues: A focus on cross-talk between molybdenum and other nutrients. J. Exp. Bot..

[29] Olsen L.I., Palmgren M.G. (2014). Many rivers to cross: The journey of zinc from soil to seed. Front. Plant Sci..

[30] Uauy C., Distelfeld A., Fahima T., Blechl A., Dubcovsky J. (2006). A NAC gene regulating senescence improves grain protein, zinc, and iron content in wheat. Science.

[31] Wiggenhauser M., Aucour A.M., Telouk P., Blommaert H., Sarret G. (2021). Changes of cadmium storage forms and isotope ratios in rice during grain filling. Front. Plant Sci..

[32] Uraguchi S., Kamiya T., Sakamoto T., Kasai K., Sato Y., Nagamura Y., Yoshida A., Kyozuka J., Ishikawa S., Fujiwara T. (2011). Low-affinity cation transporter (OsLCT1) regulates cadmium transport into rice grains. Proc. Natl. Acad. Sci. USA.

[33] Sasaki A., Yamaji N., Mitani-Ueno N., Kashino M., Ma J.F. (2015). A node-localized transporter OsZIP3 is responsible for the preferential distribution of Zn to developing tissues in rice. Plant J..

[34] Chang T.-G., Zhu X.-G. (2017). Source–sink interaction: A century old concept under the light of modern molecular systems biology. J. Exp. Bot..

[35] Yan B.-F., Nguyen C., Pokrovsky O.S., Candaudap F., Coriou C., Bussière S., Robert T., Cornu J.Y. (2018). Contribution of remobilization to the loading of cadmium in durum wheat grains: Impact of post-anthesis nitrogen supply. Plant Soil.

[36] Counce P.A., Keisling T.C., Mitchell A.J. (2000). A Uniform, Objective, and Adaptive System for Expressing Rice Development. Crop Sci..

[37] Wu F.-l., Lin D.-y., Su D.-c. (2011). The Effect of Planting Oilseed Rape and Compost Application on Heavy Metal Forms in Soil and Cd and Pb Uptake in Rice. Agric. Sci. China.

[38] Sreenivasulu N. (2019). Rice Grain Quality.

[39] Read T.L., Doolette C.L., Cresswell T., Howell N.R., Aughterson R., Karatchevtseva I., Donner E., Kopittke P.M., Schjoerring J.K., Lombi E. (2019). Investigating the foliar uptake of zinc from conventional and nano-formulations: A methodological study. Environ. Chem..

[40] Du Y., Kopittke P.M., Noller B.N., James S.A., Harris H.H., Xu Z.P., Li P., Mulligan D.R., Huang L. (2015). In situ analysis of foliar zinc absorption and short-distance movement in fresh and hydrated leaves of tomato and citrus using synchrotron-based X-ray fluorescence microscopy. Ann. Bot..

[41] Yang J.C., Zhang J.H., Wang Z.Q., Zhu Q.S., Liu L.J. (2003). Involvement of abscisic acid and cytokinins in the senescence and remobilization of carbon reserves in wheat subjected to water stress during grain filling. Plant Cell Environ..

[42] Tetyuk O., Benning U.F., Hoffmann-Benning S. (2013). Collection and analysis of Arabidopsis phloem exudates using the EDTA-facilitated Method. J. Vis. Exp..

[43] Deng T.H.B., Tang Y.T., van der Ent A., Sterckeman T., Echevarria G., Morel J.L., Qiu R.L. (2016). Nickel translocation via the phloem in the hyperaccumulator *Noccaea caerulescens* (Brassicaceae). Plant Soil.

[44] Wu F.B., Dong J., Qian Q.Q., Zhang G.P. (2005). Subcellular distribution and chemical form of Cd and Cd-Zn interaction in different barley genotypes. Chemosphere.

[45] Buckley W.T., Buckley K.E., Huang J.J. (2010). Root cadmium desorption methods and their evaluation with compartmental modeling. New Phytol..

[46] Zhang Y., Wang X., Ji X., Liu Y., Lin Z., Lin Z., Xiao S., Peng B., Tan C., Zhang X. (2019). Effect of a novel Ca-Si composite mineral on Cd bioavailability, transport and accumulation in paddy soil-rice system. J. Environ. Manag..

[47] Sun D., Zhang X., Liao D., Yan S., Feng H., Tang Y., Cao Y., Qiu R., Ma L.Q. (2022). Novel Mycorrhiza-Specific P Transporter PvPht1;6 Contributes to As Accumulation at the Symbiotic Interface of As-Hyperaccumulator *Pteris vittata*. Environ. Sci. Technol..

[48] Shi R., Weber G., Koster J., Reza-Hajirezaei M., Zou C., Zhang F., von Wiren N. (2012). Senescence-induced iron mobilization in source leaves of barley (*Hordeum vulgare*) plants. New Phytol..

[49] Maillard A., Diquelou S., Billard V., Laine P., Garnica M., Prudent M., Garcia-Mina J.M., Yvin J.C., Ourry A. (2015). Leaf mineral nutrient remobilization during leaf senescence and modulation by nutrient deficiency. Front. Plant Sci..

[50] Marschner P.J. (2012). Marschner’s Mineral Nutrition of Higher Plants.

[51] Verbruggen N., Hermans C., Schat H. (2009). Mechanisms to cope with arsenic or cadmium excess in plants. Curr. Opin. Plant Biol..

[52] Liu J.-g., Qu P., Zhang W., Dong Y., Li L., Wang M.-x. (2014). Variations among rice cultivars in subcellular distribution of Cd: The relationship between translocation and grain accumulation. Environ. Exp. Bot..

[53] Zhang W., Lin K., Zhou J., Zhang W., Liu L., Zhang Q. (2014). Cadmium accumulation, sub-cellular distribution and chemical forms in rice seedling in the presence of sulfur. Environ. Toxicol. Pharmacol..

[54] Kuepper H., Lombi E., Zhao F.-J., McGrath S.P. (2000). Cellular compartmentation of cadmium and zinc in relation to other elements in the hyperaccumulator *Arabidopsis halleri*. Planta.

[55] Herren T., Feller U. (1996). Effect of locally increased zinc contents on zinc transport from the flag leaf lamina to the maturing grains of wheat. J. Plant Nutr..

[56] Page V., Feller U. (2005). Selective transport of zinc, manganese, nickel, cobalt and cadmium in the root system and transfer to the leaves in young wheat plants. Ann. Bot..

[57] Haslett B. (2001). Zinc Mobility in Wheat: Uptake and Distribution of Zinc Applied to Leaves or Roots. Ann. Bot..

[58] Feng X., Han L., Chao D., Liu Y., Zhang Y., Wang R., Guo J., Feng R., Xu Y., Ding Y. (2017). Ionomic and transcriptomic analysis provides new insight into the distribution and transport of cadmium and arsenic in rice. J. Hazard. Mater..

[59] Fujimaki S., Suzui N., Ishioka N.S., Kawachi N., Ito S., Chino M., Nakamura S. (2010). Tracing cadmium from culture to spikelet: Noninvasive imaging and quantitative characterization of absorption, transport, and accumulation of cadmium in an intact rice plant. Plant Physiol..

[60] Satoh-Nagasawa N., Mori M., Nakazawa N., Kawamoto T., Nagato Y., Sakurai K., Takahashi H., Watanabe A., Akagi H. (2012). Mutations in Rice (*Oryza sativa*) Heavy Metal ATPase 2 (OsHMA2) Restrict the Translocation of Zinc and Cadmium. Plant Cell Physiol..

