# Supplementary material for: Differential Effects of Senescence on the Phloem Exports of Cadmium and Zinc from Leaves to Grains in Rice during Grain Filling
Source: Plants (Basel). 2023 May 6;12(9):1902. doi: 10.3390/plants12091902 (PMC10180549; doi:10.3390/plants12091902)
Supplement: Supplementary file 1 [file plants-12-01902-s001.zip › plants-2364126-supplementary.pdf]

Supporting information

Supplementary Table S1. Amount of total Cd and Zn in each organ harvested at 6 DAA (after anthesis) and at 15 DAA (after anthesis) in rice. Values are means ± standard error of 4 independent replicates.

	Cd amount (μg)			Zn amount (μg)		
Harvest	6 DAA	15 DAA		6 DAA	15 DAA	
	6 DAA	CK	ABA	6 DAA	CK	ABA
Grains	0.00*±0.00	0.12±0.02 a	0.13±0.03 a	1.33±0.94	32.11±5.22 a	30.42±4.73 a
H+R	0.12±0.05	0.10±0.04 a	0.12±0.03 a	38.06±3.18	15.18±1.87 a	21.76±3.61 b
Flag leaves	1.53±0.19	1.65±0.11 a	1.02±0.15 b	21.82±4.8	11.11±0.81 a	13.68±3.20 a
Node-I	0.00*±0.00	0.02±0.00 a	0.01±0.00 a	6.49±0.75	11.10±0.68 a	15.98±0.53 b
Stems	0.04±0.01	0.08±0.01 a	0.08±0.03 a	65.24±10.91	49.40±6.88 a	80.64±9.71 b
Lower leaves	0.21±0.03	0.97±0.57 a	0.32±0.07 a	65.16±4.44	27.70±3.92 a	37.89±6.40 a
Other tillers	0.46±0.05	0.73±0.28 a	1.17±0.20 a	244.29±40.64	214.03±9.21 a	330.21±15.83 b
Roots	1.92±0.58	3.39±0.47 a	2.05±0.33 a	476.19±148.15	739.54±162.47 a	507.42±102.56 b
Labeling tiller	1.91±0.15	2.95±0.46 a	1.69±0.18 a	198.10±15.41	146.59±12.55 a	200.38±17.57 a
Straw	2.37±0.20	3.56±0.57 a	2.73±0.37 a	441.06±55.12	328.52±16.10 a	500.17±30.10 b
Shoots	2.37±0.20	3.68±0.58 a	2.86±0.36 a	442.39±56.06	360.62±19.12 a	530.60±28.92 b
Whole plant	4.29±0.79	7.08±1.00 a	4.92±0.56 a	918.58±92.09	1100.16±156.10 a	1038.01±160.86 a

Whole plant was the sum of the dry weight of each organ. Shoot, total aboveground biomass. Labeling tiller, total aboveground biomass of labeling tiller. Straw, the total amount of aboveground parts except grains. H+R, husk+rachis. * The value is very small, and it is 0 after two decimal places. For each element, different letters within a row indicate significant differences ($p < 0.05$).

Supplementary Table S2. Cd_{lab} and Zn_{lab} absorption in flag leaves during 24 hours. Values are means ± standard error of 4 independent replicates.

	Amount of Cd_{lab} before treatments (μg)	Amount of Zn_{lab} before treatments (μg)
CK<br>ABA	0.82±0.03	1.20±0.16

Supplementary Table S3. The amount Cd_{lab} and Zn_{lab} among the rice in two treatments at 6 DAA (after anthesis) and at 15 DAA (after anthesis). Values are means ± standard error of 4 independent replicates.

	Amount of Cd_{lab} (μg Cd_{lab} $plant^{-1}$)		
	6 DAA	15DAA	
	6 DAA	CK	ABA
Grains	0.001	0.06±0.01	0.05±0.01
H+R	*n.d.*	0.05±0.03	0.03±0.02
Node-I	0.0002	0.01±0.01	0.004±0.00
Stems	*n.d.*	0.01±0.00	0.02±0.01
Lower leaves	*n.d.*	*n.d.*	*n.d.*
Other tillers	*n.d.*	*n.d.*	*n.d.*
Roots	*n.d.*	*n.d.*	*n.d.*
Flag leaves	0.82±0.03	0.70±0.04	0.72±0.09
	Amount of Zn_{lab} (μg Zn_{lab} $plant^{-1}$)		
	6 DAA	15DAA	
	6 DAA	CK	ABA
Grains	0.0003	0.03±0.01	0.04±0.01
H+R	0.02±0.01	0.06±0.02	0.04±0.01
Node-I	0.22±0.02	0.34±0.03	0.55±0.04
Stems	0.04±0.02	0.09±0.02	0.10±0.03
Lower leaves	0.11±0.02	0.13±0.04	0.11±0.01
Other tillers	0.23±0.07	0.39±0.05	0.32±0.04
Roots	0.10±0.05	0.21±0.05	0.23±0.07
Flag leaves	1.20±0.16	0.38±0.03	0.39±0.03

H+R, husk+rachis. *n.d.* Zn_{lab} and Cd_{lab} under detection limit. *ns* stands for no significant difference.

Supplementary Table S4. Isotopic abundances of Cd and Zn (data from the isotope certificate).

isotope	^{106}Cd	^{108}Cd	^{110}Cd	^{111}Cd	^{112}Cd	^{113}Cd	^{114}Cd	^{116}Cd
Labled source (%)	0.01	0.02	1.39	97.21	1.28	0.07	0.01	0.01
Natural source (%)	1.25	0.89	12.49	12.80	24.13	12.22	28.73	7.49
isotope	^{64}Zn	^{66}Zn	^{67}Zn	^{68}Zn	^{70}Zn			
Labled source(%)	1.56	3.88	89.6	4.91	0.05			
Natural source (%)	48.63	27.90	4.10	18.75	0.62			

Supplementary Table S5. Primers for qRT-PCR.

Primer name	Primer sequence (5'to3')
F-OsLCT1	GAGTTCTTCGTCAGAGCTAC
R-OsLCT1	CAGTGCTGGATGACGAATTG
F-OsHMA2	CATAGTGAAGCTGCCTGAGATC
R-OsHMA2	GATCAAACGCATAGCAGCATCG
F-OsZIP3	GCATTGTTCAGGCTAATTTTAAGG
R-OsZIP3	GGCAGTTGAGCTATGCACATTG
F-Actin1	GGGTTCACAAGTCTGCCTATTGT
R-Actin1	ACGGGACACGACCAAGGA

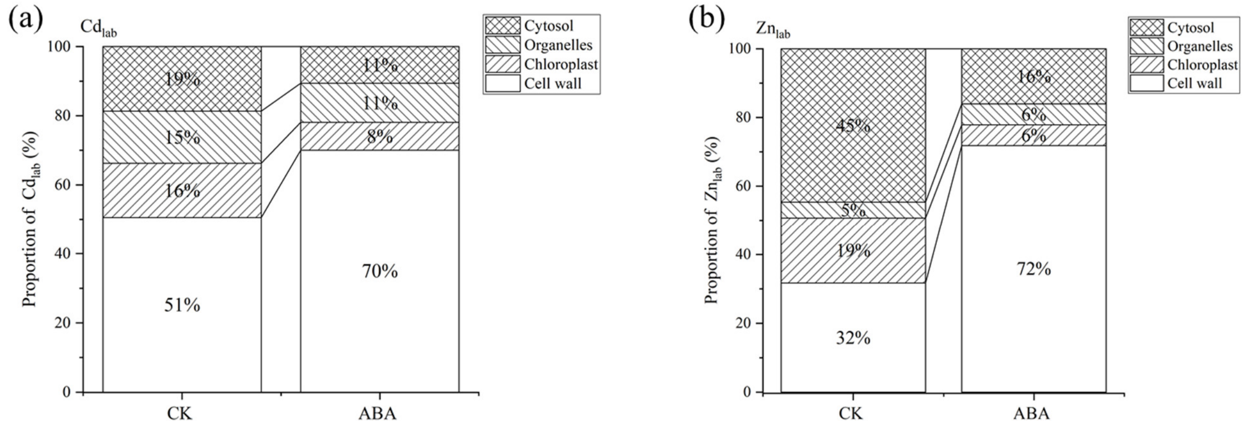

Supplementary Figure S1. Subcellular distribution proportion of Cd$_{lab}$ (a) and Zn$_{lab}$ (b) in flag leaves of rice at 15DAA (after anthesis). ABA, Abscisic Acid. CK, blank control. Cd$_{lab}$/Zn$_{lab}$, the labeled source of Cd/Zn with an enriched ^{111}Cd /^{67}Zn isotope. Data are mean values calculated from 4 independent replicates.
